# Molecular identification and subtyping of *Cryptosporidium* spp. in laboratory mice and rats[Fn FN1]

**DOI:** 10.1051/parasite/2024073

**Published:** 2024-12-04

**Authors:** Shanshan Zhou, Xinyu Hu, He Li, Zhongying Yuan, Zhen Li, Aiqin Liu, Yanyan Jiang, Jianping Cao

**Affiliations:** 1 School of Global Health, Chinese Center for Tropical Diseases Research, Shanghai Jiao Tong University School of Medicine Shanghai 200025 China; 2 National Key Laboratory of Intelligent Tracking and Forecasting for Infectious Diseases, NHC Key Laboratory on Parasite and Vector Biology, National Institute of Parasitic Diseases, Chinese Center for Disease Control and Prevention, Chinese Center for Tropical Diseases Research Shanghai 200025 China; 3 World Health Organization Centre for Tropical Diseases Shanghai 200025 China; 4 Department of Parasitology, Harbin Medical University Harbin 150081 Heilongjiang China

**Keywords:** *Cryptosporidium* spp., Zoonotic, Laboratory rodents, Genotyping, Subtyping

## Abstract

*Cryptosporidium* species can infect humans and more than 260 animal species, including 54 rodent species. However, data on the occurrence and genetic characterizations of *Cryptosporidium* spp. in laboratory rodents are limited. The present study aimed to determine the occurrence rate and genetic characterizations of *Cryptosporidium* spp. in laboratory mice and rats. We collected 506 fresh combined fecal pellet specimens (457 from mice and 49 from rats) of more than 2,000 laboratory rodents in Heilongjiang Province and Shanghai City, China. *Cryptosporidium* spp. were identified and subtyped by DNA sequencing of the SSU rRNA and the *gp60* genes, respectively. By sequence analysis of the SSU rRNA gene, the occurrence rate of *Cryptosporidium* spp. was 16.6% (84/506) in combined fecal specimens, with 18.2% (83/457) for mice and 2.0% (1/49) for rats. *Cryptosporidium parvum* (*n* = 39), *C. tyzzeri* (*n* = 33), and *C. parvum* + *C. tyzzeri* (*n* = 11) were identified in mice. *Cryptosporidium parvum* was only detected in one rat fecal specimen. At the *gp60* locus, 71.4% (60/84) of the *Cryptosporidium*-positive specimens were successfully amplified, and they all came from mice. We identified five *C. parvum* subtypes (IIaA14G2R1, IIaA16G2R1, IIaA17G1R1, IIaA17G2R1, and IIaA18G2R1) and two *C. tyzzeri* subtypes (IXaA6R1 and IXbA8). Based on the identification in laboratory mice of *C. parvum* subtypes that have been reported previously in humans, the mice infected with this species may threaten human health, especially for people who have contact with the animals and their feces.

## Abstract (fr)

Les espèces de *Cryptosporidium* peuvent infecter les humains et plus de 260 espèces animales, dont 54 espèces de rongeurs. Cependant, les données sur la présence et les caractérisations génétiques de *Cryptosporidium* spp. chez les rongeurs de laboratoire sont limitées. La présente étude visait à déterminer le taux de présence et les caractérisations génétiques de *Cryptosporidium* spp. chez des souris et des rats de laboratoire. Nous avons collecté 506 échantillons de boulettes fécales fraîches combinées (457 de souris et 49 de rats) de plus de 2 000 rongeurs de laboratoire dans la province du Heilongjiang et la ville de Shanghai, en Chine. Les *Cryptosporidium* spp. ont été identifiés et sous-typés par séquençage de l’ADN des gènes SSU rRNA et *gp60*, respectivement. Par analyse de séquence du gène SSU rRNA, le taux de présence de *Cryptosporidium* spp. était de 16,6% (84/506) dans les échantillons fécaux combinés, avec 18,2 % (83/457) pour les souris et 2,0 % (1/49) pour les rats. *Cryptosporidium parvum* (*n* = 39), *C. tyzzeri* (*n* = 33) et *C. parvum* + *C. tyzzeri* (*n* = 11) ont été identifiés chez la souris. *Cryptosporidium parvum* n’a été détecté que dans un échantillon fécal de rat. Au locus *gp60*, 71,4 % (60/84) des échantillons positifs à *Cryptosporidium* ont été amplifiés avec succès, et ils provenaient tous de souris. Nous avons identifié cinq sous-types de *C. parvum* (IIaA14G2R1, IIaA16G2R1, IIaA17G1R1, IIaA17G2R1 et IIaA18G2R1) et deux sous-types de *C. tyzzeri* (IXaA6R1 et IXbA8). Sur la base de l’identification, chez des souris de laboratoire, de sous-types de *C. parvum* qui ont déjà été signalés chez l’homme, les souris infectées par cette espèce peuvent menacer la santé humaine, en particulier pour les personnes qui sont en contact avec les animaux et leurs excréments.

## Introduction

Laboratory animals are widely used in academic and research institutions for experimental purposes. To date, at least 200 animal species – mainly rodent species, such as mice, rats, guinea pigs, and hamsters – have been used as laboratory animals [1]. Among them, mice and rats are used most commonly [27] because both of them share high genetic similarity with humans (on average, 85% of protein-coding regions are the same) and demonstrate a strong and fast reproductive capacity, easy inbred strain cultivability, and low breeding cost. According to the annual reports of the United States Department of Agriculture, mice and rats account for 99.3% (range = 97.3%–99.9%; median = 99.4%) of mammals used in laboratories annually [5]. To ensure the welfare of laboratory animals and obtain reliable experimental data, it is necessary to monitor their health status and well-being. In recent years, *Cryptosporidium* spp. were detected in laboratory mice and rats in several studies [2, 21, 24, 26, 40]. *Cryptosporidium* spp. are important intestinal protozoa infecting a broad variety of hosts, including humans [34]. The fact that some species/genotypes of *Cryptosporidium* spp. are found in both humans and animals reflects their zoonotic nature [32].

*Cryptosporidium* oocysts excreted by infected hosts are immediately infectious to other hosts. Human *Cryptosporidium* infections spread through fecal-oral transmission, either directly (person-to-person and animal-to-person transmission) or indirectly (waterborne and foodborne transmission) [31]. Due to the wide range of animal hosts and the huge number of animals, animal-to-person transmission has been attracting more and more concern. To date, more than 20 outbreaks related to contact with animals have been reported [22], mainly occurring among veterinarians and veterinary students as well as other people exposed to livestock and children visiting farms [4, 14, 18, 20, 30, 33]. In an academic research laboratory in the Unites States, one cryptosporidiosis outbreak occurred among workers caring for pre-weaned calves [12]. *Cryptosporidium* spp. primarily invade the epithelial cells of the small intestine of infected hosts and cause a disease mainly characterized by diarrhea. Most seriously, in patients with HIV/AIDS or immunocompromized individuals, diarrhea caused by *Cryptosporidium* spp. can become chronic or life-threatening [17].

*Cryptosporidium* is a complex genus, with extensive genetic variations. To date, at least 49 *Cryptosporidium* species and approximately 120 genotypes have been recognized based on the small-subunit ribosomal RNA (SSU rRNA) gene [15, 38], with 23 species and two genotypes being found in humans [9, 19]. Recently, it was noted that *C. mortiferum*, which is found in rodents (particularly, but not only, squirrels), has been commonly identified in human cases of *Cryptosporidium* infection in many countries of Scandinavia (Norway, Sweden, and Finland), raising concern about this emerging zoonotic species of importance [37, 38]. Since the first report of *Cryptosporidium* spp. in the peptic glands of tame mice in 1907 [39], epidemiologic studies have documented at least 25 *Cryptosporidium* species and 43 genotypes in 54 rodent species [41]. Limited studies have detected five *Cryptosporidium* species (*C. parvum*, *C. ubiquitum*, *C. andersoni*, *C. muris*, and *C. tyzzeri*) and one genotype (rat genotype II) in laboratory mice and rats [2, 21, 24, 26, 40]. Molecular epidemiologic data of human cases of cryptosporidiosis have confirmed *C. parvum* and *C. ubiquitum* as common species, while *C. andersoni* and *C. muris* are minor species, and *C. tyzzeri* is a rare species [9]. The establishment of subtyping tools targeting the 60-kDa glycoprotein (*gp60*) gene and the application of the *gp60* nomenclature system have substantially enhanced our understanding of the transmission of *Cryptosporidium* spp., mainly tracing the source of infection and inferring the route of transmission more accurately [25, 32]. In the present study, we assessed the occurrence rate and genetic characterizations of *Cryptosporidium* spp. in laboratory mice and rats at both genotype and subtype levels.

## Materials and methods

### Ethics statement

Fecal specimens of laboratory mice and rats were obtained from three medical experimental animal centers (MEACs) after obtaining permission from the director of each MEAC. The animal care and experimental procedures complied with the Chinese Laboratory Animal Administration Act (2017), and no animals were harmed during specimen collection. The objectives and protocols of the present study were reviewed and approved by the Laboratory Animal Welfare & Ethics Committee (LAWEC) of the National Institute of Parasitic Diseases, Chinese Center for Disease Control and Prevention, China (reference no. IPD-2021-21).

### Specimen collection

During a period of 5 months (April to August 2023), we collected fecal specimens from > 2,000 laboratory mice and rats from three MEACs, including MEAC1 and MEAC2 in Heilongjiang (China) and MEAC3 in Shanghai (China). In total, 506 combined fecal specimens (approximately 2 g per specimen from 110 to 130 mouse fecal pellets or 9 to 11 rat fecal pellets) were collected by arbitrarily selecting fresh fecal pellets from laboratory animal cages (no. of the mice per cage = 5–7; no. of the rats per cage = 3–5). Among them, 457 mouse fecal specimens (390 from Heilongjiang and 67 from Shanghai) and 49 rat fecal specimens (45 from Heilongjiang and four from Shanghai) were collected (Table 1). Gender, age, strain, and health status were not recorded during sampling.

**Table 1:** Molecular identification of *Cryptosporidium* spp. in laboratory mice and rats.

Rodent species	Sampling site	Positive no./Examined no. (%)	*Cryptosporidium* species (n)		
			*C. parvum*	*C. tyzzeri*	*C. parvum + C. tyzzeri*
Mouse	MEAC1	55/244 (22.5)	39	8	8
	MEAC2	21/146 (14.4)	–	18	3
	MEAC3	7/67 (10.5)	–	7	–
	Subtotal	83/457 (18.2)	39	33	11
Rat	MEAC1	1/24 (4.2)	1	–	–
	MEAC2	0/21 (0)	–	–	–
	MEAC3	0/4 (0)	–	–	–
	Subtotal	1/49 (2.0)	1	–	–
Total		84/506 (16.6)	40	33	11

The bar “–” denotes a negative result or a failure in PCR amplification.

### DNA extraction

Each fecal specimen was homogenized in distilled water. A QIAamp Fast DNA Stool Mini Kit (QIAGEN, Hilden, Germany) was used to extract genomic DNA from approximately 200 mg of each processed specimen, according to the manufacturer’s instructions. The DNA preparations were stored at −80 °C before polymerase chain reaction (PCR) analysis.

### Genotyping and subtyping *Cryptosporidium* spp.

*Cryptosporidium* species were identified by nested PCR amplification and sequence analysis of the partial SSU rRNA gene (~830 bp) [16]. *Cryptosporidium*-positive specimens were further subtyped by nested PCR amplification and sequence analysis of the partial *gp60* gene (~400 bp) [35]. Each DNA specimen was amplified in three PCR reactions. A negative control (DNase-free water) and a positive control (*C. baileyi* DNA for the SSU rRNA gene or *C. hominis* DNA for the *gp60* gene) were included in each PCR test. 2 × TransTaq^®^–T PCR SuperMix (+dye) (TransGen Biotech Co., Beijing, China) was used for all the PCR reactions. All the secondary PCR products were analysed electrophoretically in 1.5% agarose gel dyed with GelStrain (TransGen Biotech, Beijing, China) and observed, photographed, and recorded on a Gel Doc™ XR + Imaging System (Bio-Rad, Hercules, CA, USA).

### Sequencing and analysing

All secondary PCR products of the expected size were sequenced with their respective secondary PCR primers at Shanghai Saiheng Biotechnology Co. Ltd. (Shanghai, China) by an ABI 3730XL Genetic Analyzer (Applied Biosystems, Foster City, CA, USA). The accuracy of the sequencing data was confirmed by bidirectional sequencing. Each individual sequence chromatogram was examined using the software Chromas 2.6.6 (https://technelysium.com.au/wp/chromas). For high-quality chromatograms, the forward and reverse sequences were manually assembled and aligned with each other to create a contig using the software MEGA 7 (http://www.megasoftware.net/). The obtained contigs were used to determine *Cryptosporidium* species and subtypes by comparison with reference sequences retrieved from GenBank databases (http://www.ncbi.nlm.nih.gov). For low-quality chromatograms, such as double peaks and gaps, specimens were re-amplified under optimised PCR conditions, including increasing annealing temperatures, adjusting primer concentrations and diluting DNA template.

### Nucleotide sequence accession numbers

The novel nucleotide sequences obtained in the present study were deposited in the GenBank database under the following accession numbers: PP124619 to PP124630 (SSU rRNA gene) and PP115548 to PP115564 (*gp60* gene).

## Results

### Occurrence of *Cryptosporidium* spp.

**Figure S1.**
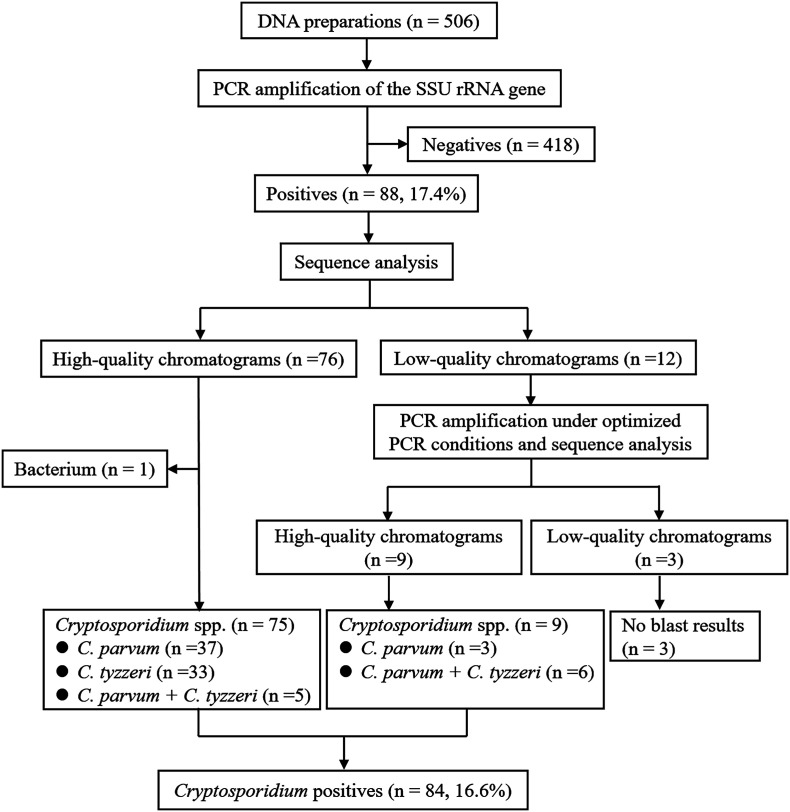
Flow chart demonstrating the molecular identification of *Cryptosporidium* spp.

At the SSU rRNA locus, 88 (17.4%) of 506 combined fecal specimens were successfully amplified. 76 specimens had high-quality DNA chromatograms; however, only 75 were determined to be infected with *Cryptosporidium* spp. based on sequence analysis. Among the remaining 12 specimens with low-quality DNA chromatograms, only nine were confirmed to be *Cryptosporidium* infections by optimising PCR conditions. In the end, a total of 84 combined fecal specimens were positive for *Cryptosporidium* spp., with an average occurrence rate of 16.6% (84/506) (Fig. S1). For mice, 18.2% (83/457) were *Cryptosporidium*-positive. *Cryptosporidium* spp. were detected from all three MEACs; in particular, the occurrence rates were 22.5% (55/244), 14.4% (21/146), and 10.5% (7/67) at MEAC1, MEAC2, and MEAC3, respectively. In contrast, *Cryptosporidium* spp. were detected in only one rat fecal specimen from MEAC1 (Table 1).

### *Cryptosporidium* genotyping and subtyping

At the SSU rRNA locus, sequence analysis identified two *Cryptosporidium* species in 84 *Cryptosporidium*-positive specimens: *C. parvum* (*n* = 40), *C. tyzzeri* (*n* = 33), and *C. parvum* + *C. tyzzeri* (*n* = 11) (Table 1). Additionally, *C. parvum* and *C. tyzzeri* were found in both MEAC1 and MEAC2, with co-infection of the two species (MEAC1: 3.0%, 8/268; MEAC2: 1.8%, 3/167). In contrast, only *C. tyzzeri* was found in MEAC3. The *C. parvum* + *C. tyzzeri*-positive specimens accounted for 14.3% (8/56, 3/21) of the *Cryptosporidium*-positive specimens in both MEAC1 and MEAC2. In the case of *C. parvum*, 15 representative sequences were obtained out of 51 specimens (40 *C. parvum* and 11 *C. parvum* + *C. tyzzeri*-positive specimens). Among them, three sequences from 38 specimens had been published previously, while the remaining 12 from 13 specimens had not been described previously (PP124619 to PP124630). In the case of *C. tyzzeri*, all 44 sequences (33 *C. tyzzeri* and 11 *C. parvum* + *C. tyzzeri*-positive specimens) were identical to each other. Detailed results of the homology analysis of the SSU rRNA gene sequences of *Cryptosporidium*-positive specimens are shown in Table S1.

At the *gp60* locus, 71.4% (60/84) of the *Cryptosporidium*-positive specimens were successfully amplified and subtyped, with 42.5% (17/40), 97.0% (32/33), and 100% (11/11) of *C. parvum*, *C. tyzzeri*, and *C. parvum* + *C. tyzzeri* specimens, respectively (Table 2). Five *C. parvum* subtypes were identified out of 12 unique sequences by analyzing 23 *gp60* gene sequences (17 *C. parvum* and six *C. parvum* + *C. tyzzeri*-positive specimens): IIaA17G2R1 (*n* = 18), IIaA16G2R1 (*n* = 2), IIaA14G2R1 (*n* = 1), IIaA17G1R1 (*n* = 1), and IIaA18G2R1 (*n* = 1). Two *C. tyzzeri* subtypes were identified out of eight distinct sequences by analyzing 40 *gp60* gene sequences (32 *C. tyzzeri* and eight *C. parvum* + *C. tyzzeri*-positive specimens): IXaA6R1 (*n* = 34) and IXbA8 (*n* = 6). Detailed results of homology analysis of the *gp60* gene sequences of *Cryptosporidium*-positive specimens are shown in Table S2.

**Table 2:** Subtyping of *Cryptosporidium* spp. in laboratory mice and rats at the *gp60* locus.

Species (*n*)	Positive no. (amplification rate)	Subtype (*n*)
*C. parvum* (40)	17 (42.5%)	IIaA17G2R1 (14); IIaA16G2R1 (2);
		IIaA17G1R1 (1)
*C. tyzzeri* (33)	32 (97.0%)	IXaA6R1 (26); IXbA8 (6)
*C. parvum + C. tyzzeri* (11)	11 (100%)	IXaA6R1 (5); IIaA17G2R1 (3); IIaA14G2R1
		+ IXaA6R1 (1); IIaA17G2R1 + IXaA6R1 (1);
		IIaA18G2R1 + IXaA6R1 (1)
Total (84)	60 (71.4%)	IXaA6R1 (31); IIaA17G2R1 (17); IXbA8 (6);
		IIaA16G2R1 (2); IIaA17G1R1 (1);
		IIaA14G2R1 + IXaA6R1 (1); IIaA17G2R1 +
		IXaA6R1 (1); IIaA18G2R1 + IXaA6R1 (1)

## Discussion

*Cryptosporidium* spp. are common intestinal protozoa infecting rodents. However, only a few studies are available on *Cryptosporidium* infections in laboratory rodents. In the present study, the overall occurrence rate of *Cryptosporidium* spp. in laboratory mice and rats was 16.6%, which was higher than those reported in three Chinese studies (1.7%–4.3% in mice and 0.6%–4.0% in rats) and one Nigerian study (1.5% in rats), but lower than that in mice in a Turkish study (100%) [2, 21, 24, 26, 40]. In our study, the higher *Cryptosporidium* occurrence rate might be attributable to sample pooling from each cage, as a single positive specimen could represent as many as seven animals, not all of which are necessarily shedding oocysts.

In the present study, based on sequence analysis of the SSU rRNA gene, *C. parvum* and *C. tyzzeri* were identified in mice fecal specimens, while *C. parvum* was detected in one rat fecal specimen. Two and four molecular studies of *Cryptosporidium* spp. have been conducted in laboratory mice and rats, respectively [2, 21, 24, 40]. All relevant studies to date, including the present study, have identified three and five *Cryptosporidium* species/genotypes in mice and rats, respectively: *C. tyzzeri* (*n* = 68), *C. parvum* (*n* = 48), and *C. muris* (*n* = 4) as well as *C. tyzzeri* + *C. parvum* (*n* = 11) in mice (*n* = 131) [24, 40]; *C. tyzzeri* (*n* = 2), *C. parvum* (*n* = 1), *C. ubiquitum* (*n* = 1), *C. andersoni* (*n* = 1), and rat genotype II (*n* = 1) in rats (*n* = 6) [2, 21, 24, 40]. All these *Cryptosporidium* species, except rat genotype II, have also been found in humans [41], suggesting a potential risk of cryptosporidiosis transmission from laboratory mice and rats to humans. In this study, co-infection of *C. parvum* and *C. tyzzeri* was observed in MEAC1 and MEAC2 and accounted for 14.3% of the *Cryptosporidium*-positive specimens in both MEAC1 and MEAC2. Only *C. tyzzeri* was found in MEAC3. The reasons behind the results remained unclear, which might be related to the number of investigated specimens.

At the *gp60* locus, only 71.4% of the *Cryptosporidium*-positive specimens were successfully amplified. Meanwhile, *C. tyzzeri* was observed to have a higher PCR amplification rate than *C. parvum*. This might be related to the genetic variations within *Cryptosporidium* species. In the present study, *C. parvum* showed more intra-genetic variations than *C. tyzzeri*. We obtained more representative sequences of *C. parvum* than those of *C. tyzzeri* at the SSU rRNA and the *gp60* loci. A similar finding was reported in a genotyping and subtyping study of *Cryptosporidium* spp. in snake fecal specimens conducted in Brazil, with a PCR amplification rate of 75.0% for *C. tyzzeri* and 42.9% for *C. parvum* at the *gp60* locus [23]. The lower amplification rate of *C. parvum*-positive specimens at the *gp60* locus might be related to the low number of oocysts in some fecal specimens.

*Cryptosporidium parvum* is one of the two most common species reported in human cases of cryptosporidiosis, and it was found to be the dominant *Cryptosporidium* species in rodents (39.2%; 1801/4589) [41]. Meanwhile, sequence analysis of the *gp60* gene has identified 16 subtypes in six subtype families (IIa, IIc, IId, IIi, IIo, and IIp) in rodent-derived *C. parvum* isolates [7–8, 36, 41]. In the present study, five subtypes belonged to the subtype family IIa, and they all came from mice. Subtype IIaA17G2R1 has also been found in urban rats in Malaysia [36]. The other four subtypes (IIaA14G2R1, IIaA16G2R1, IIaA17G1R1, and IIaA18G2R1) were identified in rodents for the first time. Notably, all the above five subtypes have also been documented in humans [3, 13], implying the zoonotic potential for individuals in close contact with laboratory mice, particularly breeders, researchers, and workers.

*Cryptosporidium tyzzeri*, *Cryptosporidium* mouse genotype I previously, was formally described as a new species in 2012 [29]. Of note, *C. tyzzeri* was originally detected in laboratory mice and is mostly detected in domestic mice and small rodents, such as wood mice, brown rats, and bank voles [41]. With the accumulation of epidemiologic data on *Cryptosporidium* spp., it was also found in humans and some other non-specific animal hosts, such as pandas, black leopards, horses, and snakes [41]. Based on sequence analysis of the *gp60* gene, to date, there have been five subtypes identified, and they belonged to three subtype families (IXa–IXc) in rodent-derived *C. tyzzeri* isolates, including IXaA6R1, IXaA6R2, IXaA8, IXbA6, and IXcA6 [6, 10, 24]. The present study identified two subtypes (IXaA6R1 and IXbA8) of *C. tyzzeri*. Interestingly, *C. tyzzeri* subtypes have also been identified in some sporadic human cases of *Cryptosporidium* infection: a child with gastrointestinal symptoms in Kuwait (subtype IXaA6R2) [35], a symptomatic 25-year-old woman in the Czech Republic (subtype IXaA8) [28], and three human patients in New Zealand (subtype family IXb) [11]. Due to limited data on *gp60* sequences of *C. tyzzeri*, the zoonotic potential of the subtypes IXaA6R1 and IXbA8 needs further investigation.

In this study, we found *Cryptosporidium* spp. in laboratory mice and rats. Since breeding and experimental work conducted with these animals was performed in Chinese MEACs according to two Chinese national standards: Laboratory animal – Environment and housing facilities (GB 14925-2023) and Laboratories – General Requirements for biosafety (GB 19489-2008), we speculated that *Cryptosporidium* infections in these animals might be related to rodent chows obtained commercially. The chows are disinfected before leaving the factory. However, they might be contaminated with *Cryptosporidium* oocysts during storage (they are actually assumed to be free from contaminants when used). Currently, *Cryptosporidium* spp. contamination sources and transmission routes in these laboratory animals remain unclear. Thus, it is also necessary to investigate *Cryptosporidium* spp. in environmental specimens. To uphold the quality of laboratory animals and enhance the accuracy of experimental outcomes, the detection of *Cryptosporidium* spp. should be routinely implemented in laboratory animals. These animals positive for *Cryptosporidium* spp., even if asymptomatic, should be removed to mitigate any potential influence of *Cryptosporidium* infections on the experimental results.

## Conclusion

We demonstrated the occurrence (16.6%) of *Cryptosporidium* spp. in laboratory mice and rats, revealing the presence of *C. parvum* and *C. tyzzeri*. Five *C. parvum* subtypes and two *C. tyzzeri* subtypes were detected, and five of them were detected in rodents for the first time. All identified *C. parvum* subtypes may pose a zoonotic threat to individuals in close contact with laboratory mice and their feces. As a result, individuals should exercise caution and implement appropriate safety measures when handling them.

## Supplementary material

The supplementary material for this article can be found at https://www.parasite-journal.org/10.1051/parasite/2024073/olm. *Table S1.* Homology analysis of the SSU rRNA gene sequences of *Cryptosporidium*-positive specimens. *Table S2.* Homology analysis of the *gp60* gene sequences of *Cryptosporidium*-positive specimens.
