# Supplementary material for: Molecular identification and subtyping of Cryptosporidium spp. in laboratory mice and rats
Source: Parasite. 2024 Dec 4;31:75. doi: 10.1051/parasite/2024073 (PMC11620727; doi:10.1051/parasite/2024073)
Supplement: Supplementary file 1 — Table S1. Homology analysis of the SSU rRNA gene sequences of Cryptosporidium-positive specimens. [file parasite-31-75-s1.pdf]

Table S1. Homology analysis of the *SSU rRNA* gene sequences of *Cryptosporidium*-positive specimens.

Species (n)	Accession No.[a] (host)	Accession No.[b] (n)	Homology (%)
C. parvum (51)	MT002720 (cattle); OK425871 (cattle)	— (26)	100
	MN557154 (lynx)	— (1)	100
	MT374186 (cattle); OP102684 (mice); MK982463 (human); MT043934 (goat);	— (11)	100
	MT071829 (pig); MK731971 (donkey); MN696801 (dog); MK770626 (horse);	PP124619 (1)	99.6
	MN235857 (mink); MK491508 (camel)	PP124620 (1)	99.9
		PP124621 (1)	99.9
		PP124622 (2)	99.9
		PP124623 (1)	99.9
		PP124624 (1)	99.9
		PP124625 (1)	99.9
		PP124626 (1)	99.9
		PP124627 (1)	99.9
		PP124628 (1)	99.9
		PP124629 (1)	99.9
	KF128753 (yak)	PP124630 (1)	99.9
C. tyzzeri (44)	MH913043 (yellow-necked mouse); OP102685 (rat); KM199845 (house mouse);	— (44)	100
	MT648483 (human); EU553589 (lizard); KM870601 (snake)		

The bar "—" denotes the sequence published.

[a]Accession Nos. representing the published sequences with the largest homology with the sequences obtained in the present study.

[b]Accession Nos. representing sequences obtained in the present study for the first time.
