# Supplementary material for: Molecular identification and subtyping of Cryptosporidium spp. in laboratory mice and rats
Source: Parasite. 2024 Dec 4;31:75. doi: 10.1051/parasite/2024073 (PMC11620727; doi:10.1051/parasite/2024073)
Supplement: Supplementary file 2 — Table S2. Homology analysis of the gp60 gene sequences of Cryptosporidium-positive specimens. [file parasite-31-75-s2.pdf]

Table S2. Homology analysis of the *gp60* gene sequences of *Cryptosporidium*-positive specimens.

Species (n)	Subtype (n)	Accession No.a (host)	Accession No.b (n)	Homology (%)
C. parvum (17)	IIaA17G2R1 (14)	MG738818 (camel)	— (10)	100
			PP115552 (1)	99.7
			PP115553 (1)	99.7
			PP115556 (1)	99.7
			PP115557 (1)	99.7
	IIaA16G2R1 (2)	MF142044 (calf)	PP115549 (1)	99.4
			PP115550 (1)	99.7
	IIaA17G1R1 (1)	MH796385 (human)	PP115551 (1)	99.7
C. tyzzeri (32)	IXaA6R1 (26)	GQ121030 (rodent)	— (19)	100
			PP115563 (1)	99.8
			PP115560 (6)	99.5
	IXbA8 (6)	OQ695568 (snake)	— (6)	100
C. parvum + *C. tyzzeri* (11)	IIaA17G2R1 (3)	MG738818 (camel)	— (1)	100
			PP115554 (1)	99.7
			PP115555 (1)	99.7
	IXaA6R1 (5)	GQ121030 (rodent)	— (3)	100
			PP115559 (1)	99.6
			PP115564 (1)	99.8
	IIaA17G2R1 + IXaA6R1 (1)	MG738818 (camel)[c]	— (1)	100
		GQ121030 (rodent)[d]	— (1)	100
	IIaA14G2R1 + IXaA6R1 (1)	MK034688 (calf)[c]	PP115548 (1)	99.6
		GQ121030 (rodent)[d]	PP115562 (1)	99.8
	IIaA18G2R1 + IXaA6R1 (1)	DQ630515 (cattle)[c]	PP115558 (1)	99.4
		GQ121030 (rodent)[d]	PP115561 (1)	99.8

The bar "—" denotes the sequence published.

[a]Accession Nos. representing the published sequences with the largest homology with the sequences obtained in the present study.

[b]Accession Nos. representing sequences obtained in the present study for the first time.

[c]Accession Nos. indicating the sequences of *C. parvum*.

[d]Accession Nos. indicating the sequences of *C. tyzzeri*.
